# Nanoscale Detonation Carbon Demonstrates Biosafety in Human Cell Culture

**DOI:** 10.3390/mi13081187

**Published:** 2022-07-27

**Authors:** Anastasia A. Malakhova, Denis K. Rybin, Alexandr A. Shtertser, Dina V. Dudina

**Affiliations:** 1 Institute of Cytology and Genetics, Siberian Branch of the Russian Academy of Sciences, Lavrentyev Ave. 10, Novosibirsk 630090, Russia; amal@bionet.nsc.ru; 2 Lavrentyev Institute of Hydrodynamics, Siberian Branch of the Russian Academy of Sciences, Lavrentyev Ave. 15, Novosibirsk 630090, Russia; rybindenis1990@gmail.com (D.K.R.); asterzer@mail.ru (A.A.S.)

**Keywords:** cytotoxicity, nanoscale detonation carbon, human cell culture

## Abstract

The production method of nanoscale detonation carbon (NDC) has recently been developed at Lavrentyev Institute of Hydrodynamics SB RAS. This method uses the reaction of acetylene with oxygen conducted in the detonation mode in fuel-rich acetylene–oxygen mixtures. The morphology and structural features of the NDC particles can be varied by changing the concentration of oxygen in the gaseous mixtures. The particles of NDC can serve as reinforcements in metal matrix composites and additives imparting electrical conductivity to polymer matrix composites. Before NDC can be considered for industrial applications, it is necessary to address the related biological safety concerns. The present work was aimed at determining the cytotoxicity of NDC. The NDC powders with two morphologies (obtained using different acetylene/oxygen ratios) were tested on HEK293A human cells. The NDC powder was added to the culture medium in concentrations ranging from 10 ng/mL to 400 μg/mL. The cell viability was determined by a colorimetric EZ4U test and a real-time cell analyzer xCELLigence. None of the NDC samples showed a cytotoxic effect. The results of this study allow us to recommend NDC as a safe and useful product for the development of advanced carbon-based and composite materials.

## 1. Introduction

In the past decades, significant progress has been made in the fabrication and property evaluation of carbon-based nanomaterials [1,2,3,4,5]. These materials show high potential for the development of nanotechnology, miniature devices, and nanomedicine. The biological safety of particulate materials is always a matter of serious concern. Before new materials with promising functional properties enter the industrial sector or our daily life, their biological safety should be tested. In medicine, carbon nanomaterials can be used as drug delivery vehicles [6]. At the same time, particulate materials may present an occupational hazard for engineers and researchers [7].

Carbon-based nanomaterials differ in the particle morphology, size, graphitization degree, and chemical composition. Ou et al. [8] discuss factors influencing the toxicity of graphene and related materials. Those factors are the lateral size, the surface structure, the presence of functional groups, impurities, and the degree of aggregation. The dose and dispersion of nanotubes in a matrix were shown to affect their toxicity level [9].

According to Wei et al. [10], oxygen-containing functional groups determine the toxicity of carbon black particles. It was suggested that epoxy, hydroxyl, and carboxyl groups are responsible for an increased bioreactivity and cytotoxicity of carbon black. Carbon nanomaterials with different geometries exhibit different cytotoxicity [11]. Single-walled carbon nanotubes were found to be toxic toward alveolar macrophages in vitro. The cytotoxicity level of different nanomaterials could be described by the following sequence (on a mass basis): single-wall carbon nanotubes > multi-walled carbon nanotubes > fullerene.

At present, the most commonly used nanoscale carbon product is carbon black [12]. The majority of industrial carbon black grades are produced by decomposition of hydrocarbons, e.g., using the furnace black process. Another possibility to produce carbon black is to use the detonation decomposition of acetylene [13,14,15,16]. This method allows obtaining high-quality carbon black, also known as acetylene black.

The detonation technologies of carbon production are further developing. In that framework, a novel carbon material, nanoscale detonation carbon (NDC), has recently been obtained at Lavrentyev Institute of Hydrodynamics SB RAS [17]. The fabrication process uses gaseous detonation in a controlled mode. The structural characteristics of the fabricated NDC have been reported in [18]. The product contains ultra-fine hollow carbon particles and/or graphene nano-sheets, depending on the concentration of oxygen in the acetylene–oxygen detonating mixture. The powders of NDC can be used as reinforcements (or for the synthesis of carbide reinforcements) in metal matrix composites and as additives imparting electrical conductivity to polymer matrix composites. Before NDC can be considered for industrial applications, it is necessary to address the biosafety concerns related to a new product. Therefore, the present work was aimed at determining the cytotoxicity effect of NDC on a human cell culture. NDC samples differing in the morphology of the particles were tested.

## 2. Materials and Methods

### 2.1. Fabrication and Characteristics of NDC

NDC samples were produced using the pulse gas-detonation device (PGDD) described in detail in [17]. Decomposition of acetylene at atmospheric pressure by detonation of the C_2_H_2_ + *k*O_2_ acetylene–oxygen mixtures at *k* < 1 makes it possible to obtain NDC and hydrogen at the same time [19]. The PGDD operates in a continuous cyclic mode with automatic cycle repetition. In each cycle (shot), the PGDD barrel is filled first with a working mixture of C_2_H_2_ + *k*O_2_. Then, a booster charge from a mixture of C_2_H_2_ + O_2_ is fed into the breech of the barrel and the spark plug initiates the booster charge, from which detonation is transferred to the working charge.

**Figure 1:**
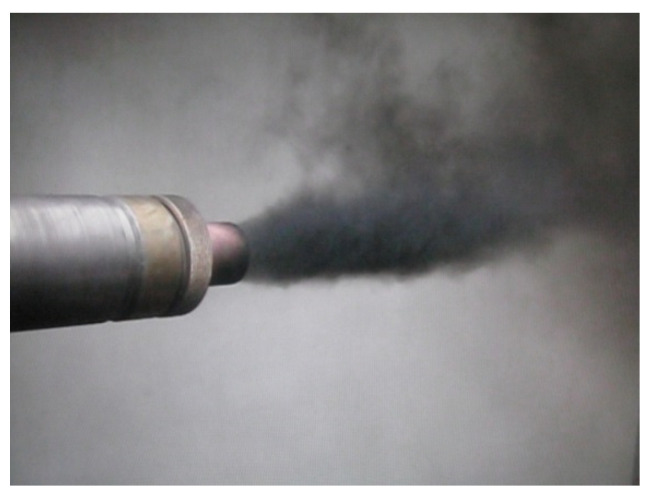
A shot of the pulse gas-detonation device (PGDD): nanoscale detonation carbon (NDC) is emitting from the barrel.

In accordance with the following reaction scheme,

C_2_H_2_ + *k*O_2_ → H_2_ + 2kCO + 2(1 − *k*)C,

the detonation products of the main charge contain nanoscale carbon, NDC. The smaller is the value of *k*, the higher is the concentration of NDC in the detonation products of the main charge. The NDC escaping from the barrel (together with the gaseous detonation products) accumulates in the product collection chamber attached to the barrel. Figure 1 illustrates the emission of NDC from the barrel during the operation of the PGDD.

**Figure 2:**
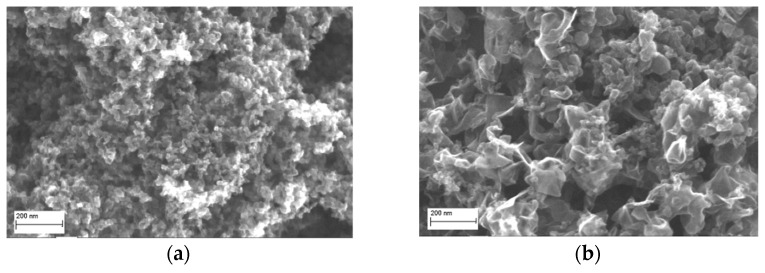
NDC produced by detonation of C_2_H_2_ + *k*O_2_ mixtures at oxygen content (**a**) 10% (*k* = 0.11) and (**b**) 40% (*k* = 0.68).

The rate of fire depends on the dimensions of the PGDD barrel. For example, when the barrel diameter is 26 mm and its length is 700 mm, the rate is 5 shots per second. The productivity of the PGDD for NDC depends on the oxygen content in the mixture and is 1.8 and 0.07 kg/h for 10% of O_2_ (*k* = 0.11) and 40% of O_2_ (*k* = 0.68), respectively [17]. Figure 2 shows the morphology of NDC produced by detonation of acetylene–oxygen mixtures at an oxygen content of 10 and 40%.

The NDC obtained at *k* = 0.11 consists of rounded particles with a size of tens of nanometers (Figure 2a), while that obtained at *k* = 0.68 consists of graphene-like particles with an in-plane size of 100–200 nm and a thickness of up to 20 nm (Figure 2b). Transmission electron microscopy studies of the carbon particles proved that the rounded particles are hollow [18]. In this work, cytotoxicity of NDC powders with these two morphologies was examined.

### 2.2. Cell Culture Cytotoxicity Assay

Cytotoxicity of the NDC samples was examined against human cell lines HEK293A (Thermo Fisher Scientific, Waltham, MA, USA) using an EZ4U colorimetric test (Biomedica, Vienna, Austria) and an xCELLigence DP Real-Time Cell Analyzer (Agilent Technologies, Santa Clara, CA, USA).

As NDC powders have poor wettability by water, dimethyl sulfoxide (DMSO) and ethanol were used to disperse the powders. NDC samples were re-suspended in DMSO (Sigma-Aldrich, St. Louis, MI, USA) or ethanol 50% in stock concentration 0.2 mg/mL. For the EZ4U test, the cells were plated onto a 96-well plate (2 × 10^4^ cells per well) in DMEM/F12 medium (Thermo Fisher Scientific, Waltham, MA, USA) 1:1, supplemented with 10% FBS (Thermo Fisher Scientific, Waltham, MA USA), 100 U/mL penicillin–streptomycin (Thermo Fisher Scientific, Waltham, MA, USA), and 1× GlutaMAX (Thermo Fisher Scientific, Waltham, MA, USA). After 24 h, when the cell monolayer reached 30–50% confluency, the NDC suspension was added to the medium. After 72 h of cell incubation, the relative amount of alive cells was determined using colorimetric EZ4U Cell Proliferation and Cytotoxicity Assay (Biomedica, Vienna, Austria), as per the manufacturer’s protocols. The absorbance value at 450 and 620 nm was measured using a Victor X3 Multilabel Reader (PerkinElmer, Waltham, MA, USA). The measurements were carried out in four parallel experiments.

For the impedance-based real-time assay, the cells were plated onto 16-well RTCA E-plates (Agilent Technologies, Santa Clara, CA, USA) (2 × 10^4^ cells per well) 24 h before adding the tested NDC samples, and the cell culture was monitored for 3 days using an xCELLigence DP Real-Time Cell Analyzer (Agilent Technologies, Santa Clara, CA, USA). The cell cultures were maintained in 5% CO_2_ atmosphere at 37 °C. Control cells were grown in the presence of 0.1% DMSO or 0.005% ethanol.

The statistical analysis was performed using ANOVA (for EZ4U) or RTCA in-app algorithm. The changes were considered significant at *p* ≤ 0.05.

## 3. Results and Discussion

### 3.1. Cytotoxicity Study of NDC Using EZ4U Assay

EZ4U is a modern improved modification of the well-known colorimetric MTT test of cell viability. The HEK293A cells were treated with different concentrations of NDC obtained at *k* = 0.11 (NDC1) and at *k* = 0.68 (NDC2). The NDC concentration ranged from 7 ng/mL to 400 μg/mL. The control cells were treated with 0.1% DMSO or 0.005% ethanol to exclude the solvent effect on the cell proliferation and viability.

**Figure 3:**
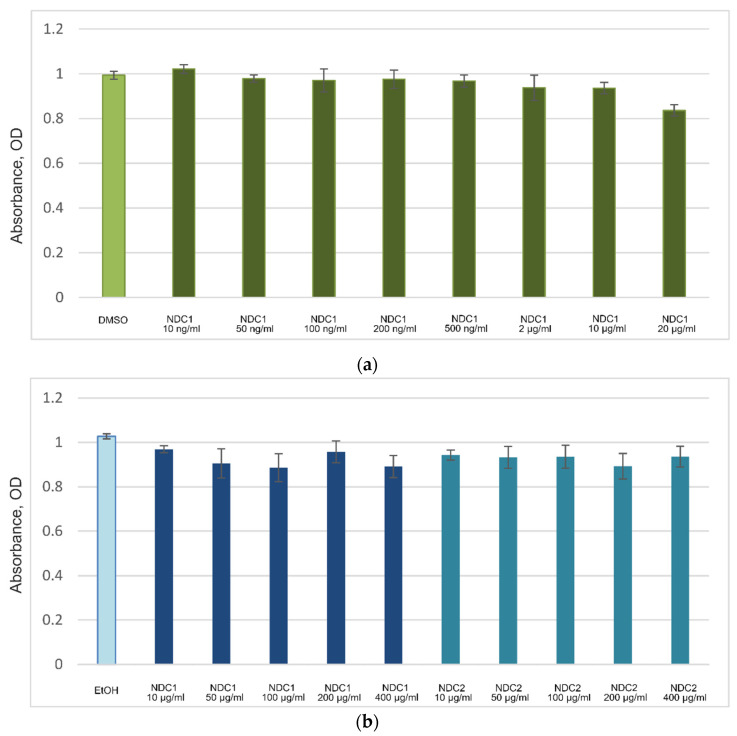
NDC effect on cell proliferation, data for different concentrations. (**a**) NDC1 re-suspended in DMSO. DMSO—solvent control. (**b**) NDC1 and NDC2 re-suspended in ethanol. EtOH—solvent control.

**Figure 4:**
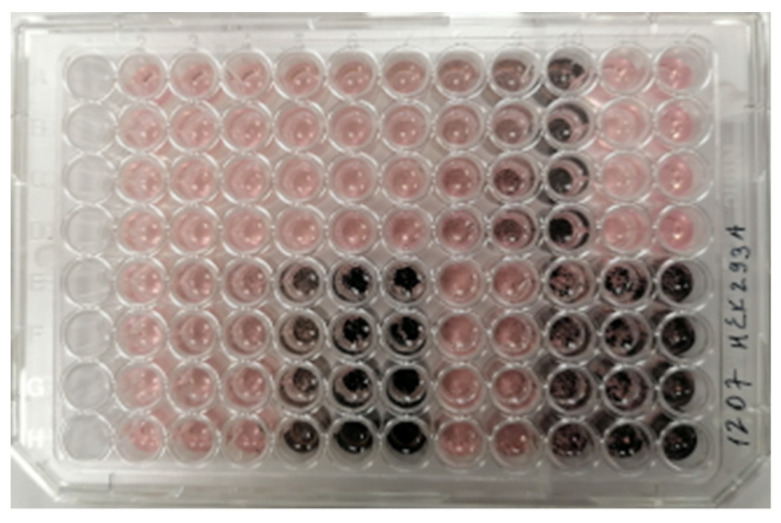
A 96-well cell culture plate with NDC samples added.

None of the NDC samples demonstrated a cytotoxic effect on the cells (Figure 3). Moreover, the carbon powder in its maximal concentration (20 μg/mL in DMSO or 400 μg/mL in ethanol) had a negligible effect (*p* > 0.05) on the cell proliferation, which could be due to the fact that NDC mechanically prevented the spread of the cells over the well surface (Figure 4).

### 3.2. Real-Time Cell Analysis Using xCELLigence

**Figure 5:**
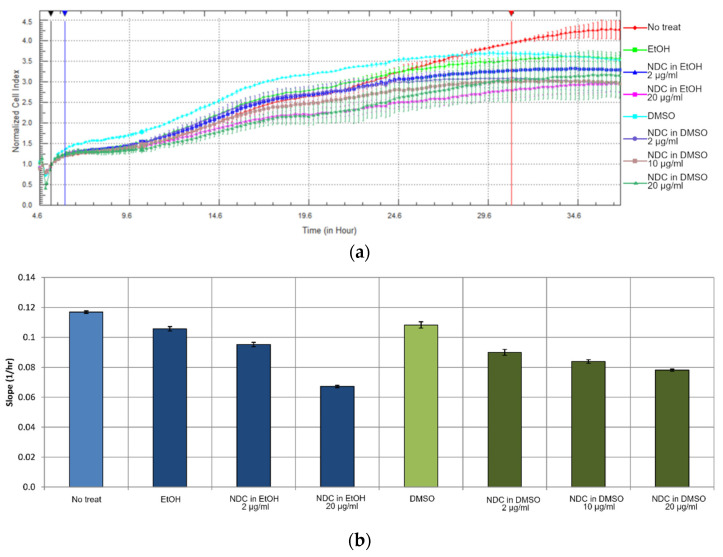
Real-time cell analysis. (**a**) Impedance-based cell growth curves. (**b**) Cell proliferation rate estimated by the growth curve slope.

For determining the effect of NDC on the cell proliferation, we performed a real-time cell analysis using an impedance-based assay. This method allows monitoring the cell proliferation in real time by measuring the culture medium impedance in special wells covered with gold electrode stripes. The real-time cell analysis was carried out with NDC1 samples (Figure 5a).

For determining the NDC effect on the cells, we analyzed the slope of the growth curves, which reflects the proliferation rate (Figure 5b). We examined the NDC influence in the first 24 h after adding the carbon samples to the growth medium. Both 0.1% DMSO and 0.005% ethanol had little effect on the cell proliferation. Only at the highest concentration (20 μg/mL) did NDC influence the proliferation rate, still being non-cytotoxic.

As noted above, the NDC obtained at *k* = 0.11 consists of rounded particles with a size of tens of nanometers. This product is similar to carbon black produced by the acetylene black process [20], in which acetylene is decomposed into carbon and hydrogen without the addition of oxygen at a high initial pressure. Carbon black has been produced for decades and no significant hazardous effects have been registered [20]. Based on long-term inhalation studies performed in rats under conditions of lung overload, the International Agency for Research on Cancer assigned carbon black to Category 2B (possible human carcinogen) [20]. It can be expected that the NDC obtained upon a small addition of oxygen at *k* = 0.11 is similar in toxicity to carbon black produced on an industrial scale. The NDC produced at *k* = 0.68 consists of graphene-like flaky particles and its biological effects may differ from those of classic carbon black and be close to those of carbon nanotubes (CNTs). According to [21], CNTs have an ability to stimulate mesenchymal cell growth and cause granuloma formation and fibrogenesis. For personnel to work with carbon nanomaterials, the content of CNTs in the ambient air should not exceed 1.0 μg/m^3^ [22]. In many cases, the health safety of new nanocarbon materials is still an open question, and studies of their biological effects should be continued.

Porous carbon materials that are biocompatible and suitable for making body implants were produced [23]. Those were graphene scaffolds prepared by spark plasma sintering. The materials had a macroporous microstructure and a high mechanical strength. Another promising direction in the application of carbon nanomaterials is the development of biologically active metal matrix composites. For example, graphene-modified Mg-based nanocomposites showed high cytocompatibility and superior osteogenic properties compared with the non-modified alloy [24]. In future research, it would be interesting to study the consolidation behavior of NDC and properties of the compacts as well as the biocompatibility of metal matrix composites reinforced with NDC.

### 3.3. Possible Applications of NDC and Its Advantages

Since NDC contains graphene-like particles, its main application may be related to the development of new ceramic or metal-based composites with improved mechanical and/or tribological properties [25]. The potential strengthening ability of graphene is due to its high mechanical performance. Having a fracture strength of 130 GPa and a Young’s modulus of 1 TPa, graphene is considered to be one of the strongest reinforcements for metal, ceramic, and polymer matrix composites [26].

It should be noted that the application range of nanocarbon materials is very broad. Namely, nanoscale carbon is used for making supercapacitors, lithium ion batteries, and solar cells [27]. In addition, the use of carbon in medicine is constantly growing [23,24,28]. The non-toxicity of NDC proved in this work is important from the point of view of safe work with this material in any of the listed applications.

In our opinion, NDC has two significant advantages over other types of nanoscale carbon. The first is the simplicity and safety of its production, as described in [17]. It should be noted that the traditional “explosive” methods of producing acetylene black [13,14,15] require compression of acetylene to pressures exceeding 10 bar. An uncontrolled explosion may occur during the production, so, complex and expensive measures are required to ensure the industrial safety. In contrast, NDC is produced from an acetylene–oxygen mixture at atmospheric pressure. For working under these conditions, the required safety measures do not differ from those taken during gas welding and cutting. Another advantage is associated with the production of composite coatings containing NDC. Our studies have shown that it is possible to apply composite coatings by detonation spraying simultaneously with the generation of NDC. Notably, studies described in [29,30] showed that detonation spraying of Al, Cu, Ni, and Ti in the in situ carbon generation mode can produce coatings with microhardness significantly higher than that of carbon-free metallic coatings. Thus, instead of obtaining a coating in three steps (obtaining NDC, mixing it with a metal powder, and thermal spraying of the powder mixture on the substrate), it is possible to produce a composite coating directly by detonation spraying in the mode with the NDC generation.

The studies described in this paper are the first in the NDC toxicity assessment. The obtained results can boost its use in industry and medicine.

## 4. Conclusions

In this work, the cytotoxicity of NDC powders was investigated for the first time. The effect of NDC with two different morphologies on HEK293A human cells was tested. The powder was added to the culture medium and the cell viability was determined by a colorimetric EZ4U test and a real-time cell analyzer xCELLigence. It was found that NDC is a non-toxic material. It is, therefore, possible to work with this material using precautions similar to those pertaining to the production of carbon black. The results of this study allow us to recommend NDC as a safe and useful product for the development of advanced carbon-based and composite materials.

## Data Availability

Not applicable.
